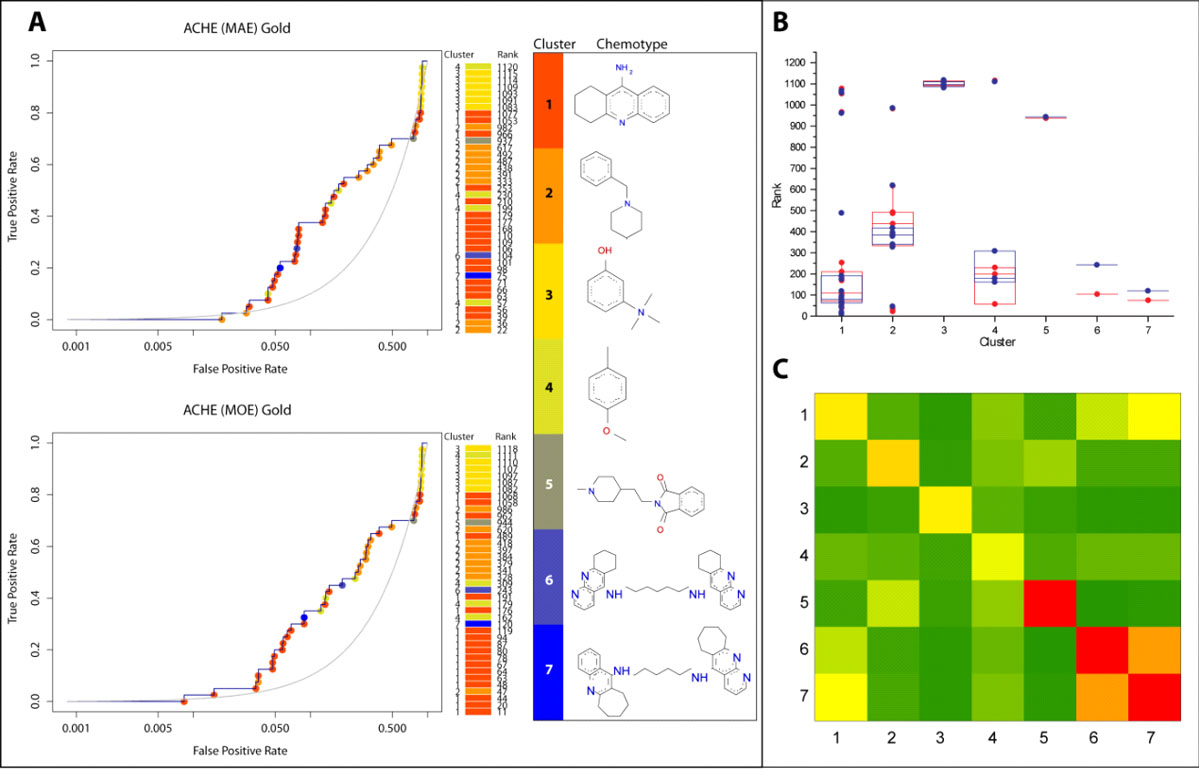# Probing the impact of protein and ligand preparation procedures on chemotype enrichment in structure-based virtual screening using DEKOIS 2.0 benchmark sets

**DOI:** 10.1186/1758-2946-6-S1-P19

**Published:** 2014-03-11

**Authors:** Tamer M Ibrahim, Matthias R Bauer, Frank M Boeckler

**Affiliations:** 1 Lab. of Molecular Design & Pharmaceutical Biophysics, University of Tuebingen, Tuebingen, 72076, Germany; 2 MRC Laboratory of Molecular Biology, Cambridge CB2 0QH, UK

## 

Structure-based virtual screening techniques can help to identify new lead structures and complement other screening approaches in drug discovery. Prior to docking, the data (protein crystal structures and ligands) should be prepared with great attention to chemistry-related molecular details. In all cases, a wide choice of commercially and non-commercially packages are available to perform such preparation schemes.

Using the DEKOIS 2.0 benchmark sets [1,2], we found differences in the respective virtual screening performance when employing different preparation schemes. We demonstrate how docking performance, particularly early enrichment, can be affected by these differences. To investigate these interesting results, we have developed an automated protocol to match and visualize ligand chemotype information in combination with the pROC profile obtained by docking. We can utilize this new tool to identify and highlight chemotype-specific behaviour, e.g. in dataset preparation. This can help to overcome chemistry-related issues in virtual screening.

**Figure 1:**